# Massively Parallel Arrays of Size‐Controlled Metallic Nanogaps with Gap‐Widths Down to the Sub‐3‐nm Level

**DOI:** 10.1002/adma.202100491

**Published:** 2021-05-03

**Authors:** Sihai Luo, Andrea Mancini, Rodrigo Berté, Bård H. Hoff, Stefan A. Maier, John C. de Mello

**Affiliations:** ^1^ Department of Chemistry Norwegian University of Science and Technology (NTNU) NO‐7491 Trondheim Norway; ^2^ Nano‐Institute Munich Faculty of Physics Ludwig‐Maximilians‐Universität München München 80539 Germany; ^3^ Blackett Laboratory, Department of Physics Imperial College London London SW7 2AZ UK

**Keywords:** lithography, molecular electronics, metallic nanostructures, nanofabrication, nanogaps

## Abstract

Metallic nanogaps (MNGs) are fundamental components of nanoscale photonic and electronic devices. However, the lack of reproducible, high‐yield fabrication methods with nanometric control over the gap‐size has hindered practical applications. A patterning technique based on molecular self‐assembly and physical peeling is reported here that allows the gap‐width to be tuned from more than 30 nm to less than 3 nm. The ability of the technique to define sub‐3‐nm gaps between dissimilar metals permits the easy fabrication of molecular rectifiers, in which conductive molecules bridge metals with differing work functions. A method is further described for fabricating massively parallel nanogap arrays containing hundreds of millions of ring‐shaped nanogaps, in which nanometric size control is maintained over large patterning areas of up to a square centimeter. The arrays exhibit strong plasmonic resonances under visible light illumination and act as high‐performance substrates for surface‐enhanced Raman spectroscopy, with high enhancement factors of up to 3 × 10^8^ relative to thin gold films. The methods described here extend the range of metallic nanostructures that can be fabricated over large areas, and are likely to find many applications in molecular electronics, plasmonics, and biosensing.

## Graphical abstract

Metallic nanogaps (MNGs) are essential elements of nanoscale devices, but the lack of reproducible, high‐yield fabrication methods has hindered practical applications. This work describes a simple, scalable approach for fabricating size‐controlled MNGs with gap‐widths as small as 3 nm. Applications of the technique include the fabrication of large‐area plasmonic substrates formed from hundreds of millions of ring‐shaped nanogaps.

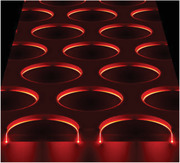

## 1 Introduction

Laterally aligned metal electrodes, separated on the nanometer length scale, are essential elements of many nanoscale photonic and electronic devices.^[^ ^1^, ^2^ ^]^ The small gap widths make them ideal choices for all‐electronic biosensors, with the capture of a single biomolecule within or across the metallic nanogap (MNG) leading to large, measurable changes in the electrical characteristics.^[^ ^3^, ^4^ ^]^ MNGs are essential components of molecular electronic devices, where conductive molecules are attached across the gap (individually or in groups) and serve as functional semiconductors in highly miniaturized rectifiers, switches, and transistors.^[^ ^1^, ^5^ ^]^ They also permit the manipulation of light via plasmonic interactions, with illumination of the nanogap inducing resonant oscillations of the free electrons inside the metal electrodes (surface plasmon polaritons; SPPs).^[^ ^6^, ^7^, ^8^ ^]^ The oscillating electrons act as electric dipoles that re‐emit light coherently at the same frequency as the incident radiation, and additionally allow a significant fraction of the electromagnetic energy from the far field to be channeled into highly confined near‐field regions within the nanogaps. These optical near fields can be many orders of magnitude higher than the incoming light, allowing the nanogaps to act as highly localized sources of light, heat, or energetic electrons for e.g. photocatalysis,^[^ ^9^ ^]^ surface‐enhanced spectroscopy,^[^ ^10^, ^11^ ^]^ nanolasers,^[^ ^12^ ^]^ solar cells,^[^ ^13^ ^]^ and plasmonic circuits.^[^ ^14^ ^]^

Nanogap devices promise to revolutionize numerous aspects of modern science and technology, but they are currently being held back by the absence of fast, controlled, reliable (high yield), and low‐cost methods of fabricating in‐plane MNGs with electrode spacings below 10 nm. Typical fabrication methods entail the use of e‐beam lithography (EBL),^[^ ^15^, ^16^, ^17^ ^]^ mechanical break junctions,^[^ ^5^, ^18^, ^19^ ^]^ electrochemical migration,^[^ ^20^, ^21^ ^]^ wet chemical methods,^[^ ^22^ ^]^ atomic layer deposition,^[^ ^23^, ^24^ ^]^ or focused‐ion beam (FIB) milling^[^ ^25^, ^26^ ^]^ to create the nanogaps (see Table S1, Supporting Information for a comparison of common nanogap fabrication techniques). Such methods, however, variously suffer from low throughput, poor scalability to large substrate sizes, complex multistep processing protocols, and/or high equipment costs. In addition, most techniques are limited to the patterning of a single metal, and so cannot be applied to the fabrication of asymmetric nanoscale devices based on dissimilar metals. The most advanced technique for patterning at the sub‐10‐nm level is extreme UV lithography (EUVL), which extends optical projection lithography to UV wavelengths of ≈13.5 nm.^[^ ^27^, ^28^ ^]^ Still in its early stages of commercialization, EUVL is available to very few researchers and as yet cannot access feature sizes below ≈5 nm or asymmetric device structures.

In several recent reports,^[^ ^23^, ^29^, ^30^, ^31^, ^32^, ^33^ ^]^ it has been shown that peeling‐based methods—which use surface modification to spatially control the adhesion of a deposited metal to underlying features on a substrate—provide a simple means of fabricating nanogap electrodes. Key advantages of peeling‐based methods include ease of implementation, parallel patterning, and scalability to large‐areas. One particularly effective peeling‐based technique known as adhesion lithography (or “a‐lith”) uses self‐assembled monolayers (SAMs) as adhesion modifiers, allowing MNGs with gap‐widths as low as 10 nm to be fabricated using a small number of simple processing steps and inexpensive equipment.^[^ ^34^, ^35^, ^36^, ^37^, ^38^, ^39^, ^40^, ^41^, ^42^, ^43^, ^44^, ^45^ ^]^ Until now, the reported gap‐widths obtained by a‐lith have been much higher than the ≈2 nm length of the SAM molecule, which is presumed to determine the absolute resolution limit of the technique. Hence, it has not been possible to apply a‐lith to applications that require the control of materials properties or light‐matter interactions at extreme (sub‐5‐nm) length‐scales, e.g. molecular electronics and plasmonics. In addition, there have been no reported methods for systematically tuning the width of the nanogaps or for fabricating massively parallel arrays of identical nanogap structures (as required e.g. for surface‐enhanced spectroscopy or catalysis^[^ ^46^ ^]^).

Herein, we introduce a new form of a‐lith that uses multilayer adhesion modifiers formed from metal‐ligated SAMs (“molecular rulers”)^[^ ^47^, ^48^, ^49^ ^]^ to improve the resolution, control, and versatility of the a‐lith procedure. The new procedure—which we call size‐tuneable adhesion lithography (STAL)—allows the gap‐width to be tuned over the range 3–30 nm (with a resolution of a few nm), and can be used to fabricate massively parallel nanogap arrays containing hundreds of millions of ring‐shaped nanogaps (RSNs) of controllable diameter, width, and pitch. Importantly STAL retains all the advantages of conventional a‐lith—namely it involves only a few simple processing steps , it uses inexpensive equipment, it can be used to fabricate nanogaps of arbitrary shape formed from dissimilar metals, and it can be applied over large areas (>1 cm^2^)—while at the same‐time providing control over the gap‐width down to the 3 nm level.

## 2 Results and Discussion

**Figure 1:**
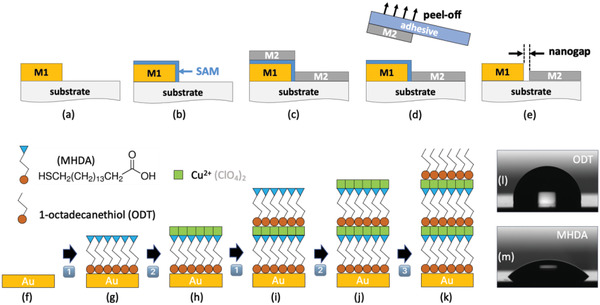
a–e) Schematic showing the key processing steps for patterning nanogaps by a‐lith and f–k) Schematic showing the key processing steps for forming size‐controllable self‐assembled multilayers. The a‐lith procedure comprises the following steps: first, a) metal M1 is deposited on a substrate and patterned as appropriate; second, b) M1 is selectively coated with a metallophilic SAM; third, c) metal M2 is deposited uniformly over M1 and the exposed substrate; fourth, d) an adhesive film is applied to the surface of M2 and the tape is then peeled away from the substrate, selectively removing M2 from those regions located directly above the SAM; finally, e) the SAM is removed by UV/ozone or oxygen‐plasma treatment, leaving M1 and M2 sitting in a complementary arrangement side‐by‐side on the substrate, separated in the limiting case by the length of the SAM. In conventional a‐lith M1 and M2 have a similar thickness, while in size‐controlled a‐lith M2 is deliberately made substantially thinner than M1 to induce fracturing of M2 along the edge‐profile of M1 (resulting in a much cleaner peel). Furthermore, in size‐controlled a‐lith, the SAM is replaced by a self‐assembled multilayer (or “molecular ruler”) formed from parallel chains of SAM molecules. f–k) The multilayer is formed by alternately immersing a gold‐coated substrate in ethanolic solutions of MHDA (Process 1) and copper perchlorate (Process 2), rinsing thoroughly in clean ethanol between each step. g,h) The first application of Process 1 yields a densely packed monolayer of MHDA, while the first application of Process 2 yields a layer of Cu(II) ions on top of the MHDA that serves as an atomically thin linker on which a second thiol SAM may be attached. i,j) Repeating the two process steps adds another SAM to the assembly, increasing the layer thickness by ≈2 nm. k) In the final step the substrate is immersed in an ethanolic solution of octadecane thiol (Process 3), yielding an alkyl‐capped upper layer. The alkyl‐functionalised SAM has a much higher water contact angle [l) 102°] than the acid‐functionalized SAM [m) 46°], leading to weaker interaction with the polar surface of the second metal and hence easier peeling. A schematic of the STAL procedure is shown in Figure S2, Supporting Information.

The a‐lith procedure is shown schematically in **Figure** **1**. In its usual form, a first metal (M1) is patterned on a substrate (Figure 1a), and conformally coated with an adhesion modifier such as an alkyl‐functionalized self‐assembled monolayer (Figure 1b), rendering it non‐adhesive to other metals; a second metal (M2) of similar height is then deposited uniformly over the full area of the substrate (Figure 1c); next, the parts of the second metal that are in contact with the adhesion modifier are stripped away using an adhesive tape or film (Figure 1d), leaving the first and second metals side‐by‐side on the substrate, with a SAM between them.^[^ ^34^, ^35^ ^]^ Finally, treatment with oxygen plasma or UV/ozone removes the SAM, resulting in a MNG (Figure 1e).

The first step toward achieving size‐tuneability is to ensure the gap‐width attained in conventional a‐lith is as close as possible to the length of the adhesion modifier, around 2 nm in the case of the 1‐octadecanethiol (ODT) SAM molecules used here.^[^ ^50^ ^]^ Successful patterning by a‐lith requires the unwanted parts of the second metal (M2) that lie above the SAM to be “split” from the (required) parts of M2 that are in direct contact with the substrate, see Figure 1d; the unwanted parts of M2 must be lifted away by the peeling layer, while the rest of M2 must remain on the substrate. To effect the split, it is necessary to overcome any cohesive forces that exist between the M2 metal atoms at the intended “break‐lines,” i.e., along the edge profile of M1. Any tearing (as opposed to clean splitting) of M2 during peeling risks widening the gap‐width undesirably. Interestingly, imaging of the surface of freshly‐deposited M2 by atomic force microscopy (AFM) has previously revealed the existence of fracture lines that follow the edge profile of M1, suggesting the necessary split has partially occurred even before the peeling step is carried out.^[^ ^34^ ^]^ In this work, to further promote this pre‐fracturing of M2, we use a staggered geometry in which we deliberately introduce a height difference between the two metals, with M2 being appreciably thinner than M1—30 nm versus 50 nm for the work reported here. The thin M2 layer is unable to conformally coat the terrain of the M1‐patterned substrate, and discontinuities in M2 therefore arise along the edge profile of M1. In other words, the height difference between the two metals causes M2 to split exactly where the wanted and unwanted parts must be separated during the peeling step, ensuring a clean peel without tearing.

**Figure 2:**
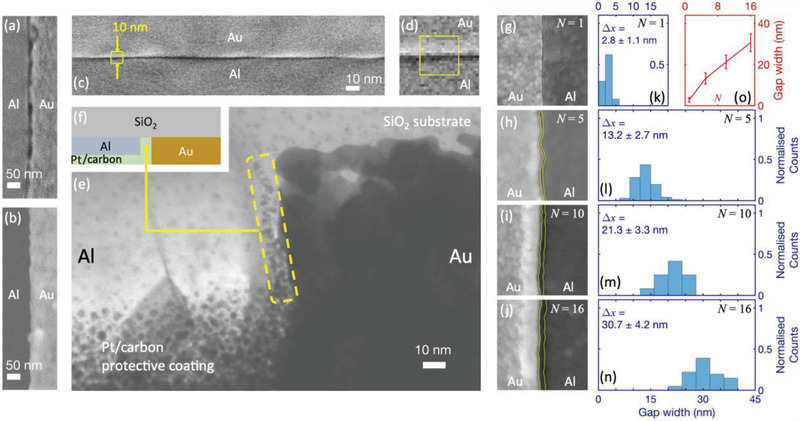
Images of nanogap electrodes formed using STAL and corresponding spacing histograms. a,b) SEM images of a Au–Al nanogap obtained with matched (a) and unmatched (b) metal heights, using a single layer of ODT as the SAM, 50‐nm Au for M1 and 50‐nm Al or 30nm Al for M2. (The image contrast in (a) has been enhanced using contrast‐limited adaptive histogram equalization for easier visualization of the gap region). c) SEM image showing a ≈240‐nm section of a 50‐nmAu/30‐nmAl single‐layernanogap obtained using a high‐resolution S(T)EM. The yellow box indicates an area of 10nm by 10nm. d) Magnified section of the SEM image from (c), showing the same 10nm by 10nm boxed region. e) TEM image of the single‐layernanogap. The dotted yellow region shows a mixture of carbon and platinum metal that has infiltrated the nanogap during sample preparation (see Methods). f) Schematic representation of the TEM image in (e). g–j) Scanning electron microscopy images of 500‐nm sections of Au/Al nanogaps obtained using molecular rulers of length *N* = 1 (g), *N* = 5 (h), *N* = 10 (i), and *N* = 16 (j). The annotated yellow lines indicate the edges of the two metals. k–n) Spacing histograms for the nanogaps in (g–j). The numerical values for the gap‐width Δ*x* represent the mean measured gap‐width plus or minus one standard deviation. o) Plot of the mean measured gap‐width versus the length *N* of the molecular ruler. The length of each error bar is twice the standard deviation of the measured gap‐width.

**Figure** **2a**,b show scanning electron microscopy (SEM) images of Au–Al nanogaps fabricated under equivalent conditions, using matched and unmatched metal heights for M1 and M2. ODT was used as the SAM, Au as the first metal, and Al as the second metal (see Experimental Section). Using a film thickness of 50 nm for both metals yielded a gap‐width of ≈20 nm at the Au/Al interface (Figure 2a), typical of a‐lith gap‐widths reported elsewhere in the literature. (Note to ease visualization of the nanogap, the image contrast in Figure 2a has been enhanced using contrast‐limited adaptive histogram equalization. The original unprocessed image may be seen in Figure S1, Supporting Information). Reducing the Al thickness to 30 nm resulted in a much narrower gap between the metals below the 3 nm resolution limit of the microscope due to pre‐fracturing of M2 before peeling (see Figure 2b). Figure 2c shows an SEM image of a Au(50 nm)/Al(30 nm) nanogap measured on a high‐resolution scanning electron microscope. The small 10 nm × 10 nm region denoted by the yellow square in Figure 2c is reproduced at higher magnification in Figure 2d. The images clearly show a very small gap between the two metals, less than 3 nm in width and close to the minimum electrode spacing that can be expected using the 2 nm ODT molecule.

Figure 2e shows a transmission electron microscopy (TEM) image of the gap. As shown schematically in Figure 2f, the upper part of the TEM image corresponds to the SiO_2_ substrate, while the lower “speckled” part on the left hand side corresponds to a carbon/platinum protective coating applied during sample preparation (see Experimental Section), which has been partially milled away to expose the Au electrode. The light region on the left between the SiO_2_ and Pt/carbon corresponds to Al, while the dark region on the right corresponds to Au. At the interface between the Al and Au, a thin strip of Pt/carbon is visible (see inside dotted yellow box), corresponding to deposited material that has penetrated the gap between the two electrodes. The speckled appearance of the Pt/carbon inside the nanogap and on top of the Al is typical of Pt/carbon deposited by FIB deposition, with the dark spots corresponding to platinum and the lighter surrounding region corresponding to carbon and other impurities.^[^ ^38^, ^51^, ^52^ ^]^ The width of the strip in the gap region indicates a sub‐3‐nm gap‐width in broad agreement with the SEM image in Figure 2c.

The original a‐lith procedure provides no method for tuning the electrode spacing, with the obtained gap‐widths typically being anywhere from five to fifty times larger than the 2 nm length of the SAM molecule (depending for instance on how “aggressively” the peeling step is carried out).^[^ ^34^, ^35^ ^]^ In the previous section, we discussed how introducing a 20 nm height difference between the two metals permits a clean split of M2 along the edge profile of M1 and so yields a gap‐width close to the 2 nm length of the SAM molecule. This suggests that, using a staggered geometry, it should be possible to tune the electrode spacing simply by changing the length of the SAM molecule or, more generally, by changing the length of the adhesion modifier.

To test this idea we replaced the SAMs used in standard a‐lith by extendable chains of metal‐ligated self‐assembled multilayers, known as molecular rulers.^[^ ^49^, ^53^ ^]^ The self‐assembled multilayers are formed using SAM molecules with thiol head groups and carboxylic acid end groups by alternately immersing a gold‐coated substrate in ethanolic solutions of the SAM molecules and copper perchlorate (see Figure 1f‐k and Experimental Section). In the first step—using Au for M1—the thiol SAM molecules are conformally attached to the patterned gold, with the carboxylic acid groups facing outward (Figure 1g). In the second step, Cu(II) ions coordinate with the carboxylic acid groups of the first SAM, forming an atomically thin layer (Figure 1h) that serves as a linker upon which a second thiol SAM may be conformally attached (Figure 1i). With each cycle, an additional SAM is added to the multilayer, increasing the layer thickness by ≈2 nm, which makes it possible to vary the length of the spacer molecules from a few nanometers to several tens of nanometers in ≈2 nm increments (see Table S2, Supporting Information).

In practice, the presence of outwardly facing carboxylic acid groups on the final layer of the molecular ruler causes unwanted interactions with the second metal (M2), preventing clean splitting and leaving unwanted “spots” of M2 on top of M1. Hence, in the final step of the multilayer deposition (Figure 1k), we replace the acid‐functionalized SAM with an alkyl SAM, resulting in a lipophilic surface that will not bind with the second metal. The significant difference in the surface properties of the two SAMs is evident in Figure 1l,m, with the alkyl‐functionalized SAM (ODT) having a substantially higher water contact angle than the acid‐functionalized SAM (102 versus 46 degrees), leading to weaker interaction with the polar surface of the evaporated aluminum. (A schematic showing the complete a‐lith procedure using molecular rulers is shown in Figure S2, Supporting Information, while Figure S3, Supporting Information, shows how the choice of an acid‐ or alkyl‐containing top layer affects the reliability of the patterning procedure).

Figure 2g–j show 500 nm x 500 nm SEM images for nanogap electrodes obtained using molecular rulers formed from *N* = 1, 5, 10, and 16 repeat units (after removal of the self‐assembled multilayer). In each case, the uppermost SAM layer was an alkyl SAM, while all other layers were acid‐functionalized. The SEM images reveal a clear increase in the spacing between the Au and the Al as the number of repeat units increases, consistent with the increasing length of the molecular spacers. Histograms showing the distribution of electrode spacings along the gap are shown adjacent to each image in Figure 2k–n, while a plot of mean gap‐width Δ*x* versus *N* is shown in Figure 2o. The mean gap‐width can be seen to vary from 2.8 nm for *N* = 1 to 30.7 nm for *N* = 16, with the relative standard deviation (defined as the ratio of the standard deviation to the mean) falling from ≈40% at 2.8 nm to ≈14% at 30.7 nm. Hence, using molecular rulers, it is possible to controllably vary the gap‐width over a range of sizes that are relevant for multiple applications in molecular sensing, molecular electronics, and plasmonics.^[^ ^4^ ^]^

**Figure 3:**
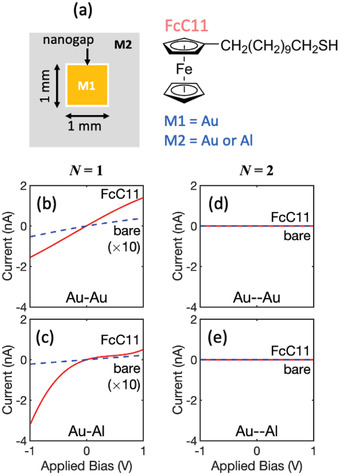
Current–voltage (*I–V*) characteristics for nanogap molecular diodes based on the conductive molecule FcC11. a) Schematic showing the geometry of the nanogap electrodes used for the measurements, with chemical structure of ferrocenyl‐1‐undecanethiol (FcC11) shown adjacent. b) *I–V* characteristics for a Au–Au nanogap device fabricated using molecular rulers of length *N* = 1, i.e. using a single layer of ODT. The dotted blue line and the solid red line show the *I–V* characteristics before and after deposition of the FcC11 monolayer. c) *I–V* characteristics for a Au–Al nanogap device fabricated using molecular rulers of length *N* = 1. d) *I–V* characteristics for a Au–Au nanogap device fabricated using molecular rulers of length *N* = 2, i.e. using a bilayer of MHDA and ODT. The measured currents are below the 10 pA detection limit of the current meter. e) *I–V* characteristics for a Au–Al nanogap device fabricated using molecular rulers of length *N* = 2. The measured currents are below the 10 pA detection limit.

To evaluate the feasibility of using the nanogap electrodes for molecular devices, arrays of square‐shaped nanogap electrodes were fabricated using Au for the first metal (M1) and either Au or Al for the second metal (M2), see Experimental Section. The first metal was deposited as a grid of 1 mm^2^ squares, and the STAL method was then applied, using an adhesion modifier formed from either one (*N* = 1) or two (*N* = 2) layers of SAM molecules, yielding square nanogaps of side‐length 1 mm (**Figure** **3**a) and respective widths ≈3 or ≈5 nm. To complete the molecular diodes, a monolayer of the widely studied molecular conductor 11‐ferrocenyl‐1‐undecanethiol (FcC11)^[^ ^54^, ^55^ ^]^ was deposited on the nanogap electrodes by immersing the electrodes overnight in a 5 × 10^−3^ m solution of FcC11 in ethanol (see Experimental Section).

The current‐voltage (*I*–*V*) characteristics of the nanogap electrodes before FcC11 deposition were measured in the bias range −1 V to +1 V, where forward bias corresponds to the second deposited metal (M2 = Au or Al) being positively biased with respect to the first deposited metal (M1 = Au). The *N* = 1 nanogap electrodes exhibited approximately linear current–voltage characteristics with high impedances of around 20 and 50 GΩ for Au/Au and Au/Al, respectively (see dotted blue lines in Figure 3b,c). In both cases the *N* = 2 nanogap electrodes exhibited currents below the ≈10 pA detection limit of the measurement, indicating extremely high impedances in excess of 100 GΩ (see dotted blue lines in Figure 3d,e). Hence, there is good electrical (and physical) isolation between the electrodes in all cases, with the *N* = 1 electrodes allowing a small measurable current to flow as a consequence of through‐air tunneling and/or possible occasional shorts between the electrodes.

The *N* = 1 nanogaps exhibited substantially higher currents after deposition of the FcC11 monolayer (see solid red lines in Figure 3b,c), indicating the FcC11 molecules are able to provide a conductive bridge between the two electrodes, with the molecules forming a covalent Au—S bond at one electrode and a van der Waals type interaction at the other.^[^ ^56^ ^]^ The Au/Au devices exhibited near‐symmetric *I*–*V* characteristics with a rectification ratio *R = |I(−1 V)/I(+1 V)|* of ≈1.1, while the Al/Au devices exhibited asymmetric characteristics with typical rectification ratios in the range 4–7. The broadly symmetric current‐voltage characteristics of the Au/Au devices are consistent with the use of identical metals for the two electrodes, and the ability of the thiol group on the FcC11 molecules to attach to both the first (M1) and second (M2) electrodes, which has the effect of averaging out effects due to the asymmetry in the molecular structure. The asymmetric current–voltage characteristics of the Au/Al devices by contrast are attributable both to the use of dissimilar metals for the two electrodes and to the thiol head groups attaching only to M1 (Au), which preserves the effects of asymmetry in the molecular structure.

The highest occupied molecular orbital (HOMO) of the ferrocene moiety lies ≈5 eV below the vacuum level,^[^ ^56^ ^],^ i.e. very close to the Fermi level of gold. Hence, in the Au/Au devices, hole transport is mediated by the HOMO level of Fc over the full bias range tested, and holes need only tunnel through the 1.3 nm length of the insulating alkyl chain for a measurable current to flow.^[^ ^55^, ^57^ ^]^ The same is true for the Au/Al devices operating under negative bias, i.e. when the Al electrode (M2) is biased negatively with respect to the Au electrode (M1). Under a moderate positive bias, by contrast, the HOMO level of Fc cannot participate in charge transport as it lies outside the Fermi levels of the electrodes,^[^ ^56^, ^58^ ^]^ meaning holes injected from the Al electrode must tunnel through the full 2 nm length of the FcC11 molecule for a current to flow. The forward bias current is therefore suppressed, and significant rectification is observed.

The solid red lines in Figure 3d,e show for Au/Au and Au/Al the measured‐current voltage characteristics of the *N* = 2 nanogap devices after application of the FcC11 molecules. In both cases, the currents remained below the 10 pA detection limit of the ammeter over the entire bias range (from −1 V to +1 V), indicating the FcC11 molecules were unable to conductively bridge the gap. For *N* = 2, we therefore conclude that the gap‐width is substantially higher than the ≈2 nm length of the FcC11 molecules over the full 4 mm length of the gap. On the other hand, for the FcC11 molecules to provide a conductive bridge between the electrodes, the *N* = 1 gap‐width must be less than 2 nm at many locations along the gap in agreement with the SEM image of Figure 2c.

**Figure 4:**
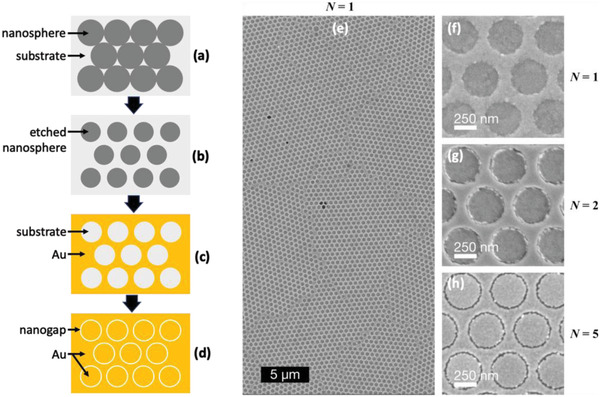
Fabrication of massively parallel nanoring arrays using a combination of nanosphere lithography and STAL. a–d) Schematic of the fabrication procedure, in which: first, a) a monolayer of close‐packed polystyrene nanospheres is deposited on a substrate; second, b) the nanospheres are “shrunk” by oxygen plasma treatment, leaving voids between them; third, c) metal M1 = Au is deposited on the substrate through the nanosphere template and the template is removed, leaving a hexagonal array of nanoholes in the Au film; and, fourth, d) the holes are “filled” with a second metal (M2 = Au) using STAL, resulting in a hexagonal array of ring‐shaped nanogaps. e) 20 µm × 40 µm SEM image of a Au–Au nanoring array, obtained using a molecular ruler of length *N* = 1. The image shows several domains of nanorings with relatively low defect densities in each domain. f–h) High magnification SEM images of Au–Au nanoring arrays, obtained using molecular rulers of length *N* = 1 (f), *N* = 2 (g), and *N* = 5 (h). Each array has a pitch of ≈500 nm and a ring‐diameter of ≈380 nm, defined by the nanosphere diameters before and after etching.

One favorable feature of a‐lith is its scalability to large areas (see, e.g., Figure S4, Supporting Information), which opens up the possibility of fabricating massively parallel arrays of nanogaps. As a proof of concept, we fabricated large‐area (≈50 mm^2^) arrays of nanorings by combining a‐lith with a soft colloidal lithography method known as nanosphere lithography (NSL).^[^ ^59^ ^]^ In brief, the combined technique comprised the following steps: a hexagonal close‐packed monolayer of polystyrene (PS) spheres (**Figure** **4a**) was deposited on a substrate by drop‐casting; the nanospheres were then isotropically “shrunk” via oxygen plasma etching so they no longer touched (Figure 4b); the first metal was evaporated onto the substrate through the spaces between the spheres; and the spheres were then removed, leaving the first metal patterned with a hexagonal array of circular holes (see Figure 4c and Figure S5a, Supporting Information); continuing the STAL method from this point (as shown in Figure 1b–e) yielded a macroscopic array of near‐identical RSNs (see Figure 4d,e and Figure S6, Supporting Information), in which the pitch, diameter, and gap‐width of the RSNs were respectively determined by the initial diameter of the PS spheres, the etched diameter of the PS spheres, and the number of layers used in the molecular ruler.

For a typical drop‐cast area of 50 mm^2^ and an initial nanosphere diameter of around 500 nm, each array contains some 200 million discrete and nominally identical nanorings (see Figure S7, Supporting Information). Like any method based on NSL, defects such as dislocations and vacancies in the initial nanosphere template introduce disorder in the final pattern. However, executed with care, the combined NSL/STAL method provides a simple means of rapidly fabricating well‐ordered and massively parallel arrays of nearly identical nanogaps that extend over multi‐millimeter length‐scales with relatively low defect densities, see, for example, Figure 4e. Figure 4f–h shows high magnification SEM images of Au/Au RSN arrays obtained using a pitch of ≈500 nm, a ring diameter of ≈380 nm, and *N* = 1, 2, or 5 layers in the molecular ruler. Clear gaps of ≈5 and ≈10 nm are evident in the SEM images for the 2‐ and 5‐layer arrays, while the gap‐width of the 1‐layer device is below the 2 nm image resolution.

It is well known that regular arrays of sub‐wavelength holes or slits in metal films allow optical energy to be efficiently coupled into SPPs—electromagnetic excitations that propagate in a wave‐like manner along the planar interface between a metal and a dielectric—or into localized surface plasmons.^[^ ^7^, ^46^, ^60^ ^]^ The confinement of the electromagnetic wave to the vicinity of the metal/dielectric interface results in a substantial enhancement of the electromagnetic field and accounts for many useful surface‐enhanced optical properties, for example increased absorption, fluorescence, Raman scattering, second‐harmonic generation, and chiroptical behavior.^[^ ^6^, ^10^, ^46^, ^60^ ^]^ The RSN arrays are therefore of potential interest for a variety of plasmonic applications.

**Figure 5:**
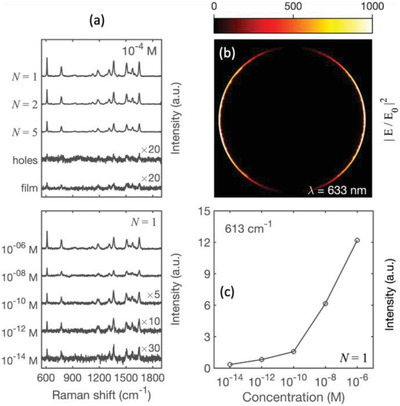
Surface‐enhanced Raman scattering from arrays of RSNs. a, top) Experimentally determined Raman scattering spectra for Rhodamine 6G (R6G) drop‐cast from a 10^−4^ m solution onto Au–Au RSN arrays fabricated using molecular rulers of length *N* = 1, 2, and 5. The arrays were fabricated with a pitch of ≈500 nm and a ring‐diameter of ≈380 nm, see Figure 4f–h. Also shown for comparison are the Raman scattering spectra for 10^−4^ m R6G drop‐cast onto a thin gold film and onto a gold nanohole array, obtained by terminating the patterning procedure at step (c) of Figure 4. All spectra were obtained under equivalent conditions with a 633 nm excitation wavelength, see Experimental Section. The spectra for the thin gold film and the nanohole array have been multiplied by a factor of 20 for clarity. a, bottom) Experimentally determined Raman scattering spectra for R6G drop‐cast onto *N* = 1 Au–Au RSN arrays from R6G dye solutions of varying concentrations. All spectra were obtained under equivalent conditions with a 633 nm excitation wavelength. The spectra for 10^−10^, 10^−12^, and 10^−14^ m R6G have been multiplied by factors of 5, 10, and 30, respectively. b) Simulated plot showing the square of the field enhancement at the height of the exposed metal surface for a ring‐shaped nanogap in the hexagonal array, assuming a gap width of Δ*r* = 3 nm (*N* = 1). c) Plot of experimentally determined scattering intensity at 613 cm^−1^ versus dye concentration, extracted from the data shown in the lower plot of (a).

To test the suitability of the RSN arrays for one such plasmonic application, their performance as surface‐enhanced Raman spectroscopy (SERS) substrates was evaluated, alongside a thin gold coating of similar thickness and a nanohole array (see Figure S5a, Supporting Information) obtained by terminating the RSN fabrication procedure after step (c) in Figure 4, i.e. before deposition of the second metal (M2). A 10^−4^ m solution of the test‐dye Rhodamine 6G (R6G) was drop‐cast on each of the five substrates, and Raman spectra were recorded under equivalent conditions at a probe wavelength of 633 nm (see Experimental Section). The resulting Raman spectra are shown in the upper plot of **Figure** **5a**. In spite of the high dye concentration, the thin Au film and nanohole array both yielded weak Raman spectra, with the characteristic Raman spectra barely visible above the noise floor of the measurements. The three RSN‐arrays, by contrast, yielded well‐defined spectra typical of R6G, with the scattering intensity increasing approximately twofold as *N* was reduced from 5 to 1.

Figure 5b shows a simulation of the square of the electromagnetic field enhancement at the top surface of the RSN array. To simulate the array we used periodic boundary conditions for a hexagonal lattice with $a\;=\sqrt{3}\;P_{x}/2$, where  *P_x_* =  500 nm is the *x* dimension of the unit cell and *a* is the apothem of the hexagon. The source employed in the simulations was a linearly polarized plane wave at λ  = 633 nm, matching the wavelength used for the SERS experiments. The gap‐size was set to Δ*r*  =  3 nm, representing the case *N*  =  1. The simulation shows high field intensity in the gap region with a spatially‐averaged field enhancement |*E*/*E*_0_|^2^ of 335, leading to high SERS enhancement for the molecules within the gap. This enhancement is achieved even though we are not operating at the exact localized plasmon resonance condition (which occurs at λ  =  626 nm, see Figure S8, Supporting Information). Indeed, a key advantage of the nanoring geometry is that the resonance peaks are very broad, which means appreciable field enhancement can be achieved without needing to precisely tune the excitation wavelength to the peak of the plasmon resonance.^[^ ^61^, ^62^ ^]^

The lower plot of Figure 5a shows a series of Raman spectra obtained using *N* = 1 RSN arrays at various dye concentrations from 10^–6^ m down to 10^–14^ m. The spectra all show the characteristic Raman scattering peaks of R6G, albeit with a reduction in scattering intensity as the dye concentration is reduced. The intensity of the strongest 633 nm peak versus dye concentration is shown for illustrative purposes in Figure 5c. The strongly sub‐linear variation of scattering intensity with dye concentration is consistent with previous reports in the literature using plasmonic substrates.^[^ ^18^, ^63^, ^64^ ^]^ Even at the lowest concentration of 10^–14^ m, most of the Raman peaks of R6G are evident. Assuming the dye molecules are randomly distributed on the RSN array and that they can couple to localized electric fields only if their centers lie within the nanogap, a purely geometric argument indicates that the measured scattering signal at 10^–14^ m is due to around 1000 dye molecules (see Figure S7, Supporting Information). Comparing the 613 cm^–1^ signal at 10^–14^ m with the signal obtained under equivalent conditions on a thin gold film at 10^–5^ m (the lowest concentration at which a signal could be measured, see Figure S9, Supporting Information) indicates an analytical enhancement factor^[^ ^65^ ^]^ of 3 × 10^8^, one of the highest values so far reported for R6G on a SERS substrate (see Table S3, Supporting Information), even though the 633 nm excitation wavelength was not tuned to be in exact resonance with the nanogap array.

## 3 Conclusion

We have reported methods for improving the resolution, control, and versatility of the a‐lith procedure. By introducing a height difference of around 20 nm between the first and second metals we were able to achieve a substantial narrowing of the achievable gap‐width to below 3 nm—close to the 2 nm length of the SAM molecule, and sufficiently small for the fabrication of molecular rectifiers using the molecular conductor 11‐ferrocenyl‐1‐undecanethiol, FcC11. Further, by replacing the fixed‐length SAMs by variable‐length chains of carboxylic acid functionalized SAM molecules (“molecular rulers”), we showed that it is possible to vary the gap‐width over the range of 2–30 nm. Finally, by combining a‐lith with NSL, i.e. using a hexagonal array of etched nanospheres as a sacrificial shadow mask for deposition of the first metal—we fabricated massively parallel arrays of RSNs of variable diameter, width, and pitch. The arrays—which extend over millimeter length‐scales and contain hundreds of millions of RSNs—strongly confine electromagnetic fields under visible light illumination, making them suitable for a range of plasmonic applications. The RSN arrays were evaluated here as substrates for SERS, using R6G as a test molecule, and were found to exhibit very high enhancement factors of 3 × 10^8^ relative to a similar (gap‐free) thin gold film.

## 4 Experimental Section

### Fabrication of Single‐Layer Metal Nanogaps

A 5 nm adhesion layer of Al followed by a 45 nm layer of Au (M1) was deposited on a glass substrate, using e‐beam deposition via a shadow mask (10^–7^ mbar, 2 Ås^−1^). The substrate was then immersed in a 2 × 10^−3^ m ethanolic solution of ODT for 24 h, before washing thoroughly in clean ethanol. A 30 nm layer of Al or Au (M2) was then deposited by e‐beam deposition over the full area of the metal‐coated substrate (10^–7^ mbar, 2 Ås^–1^). An adhesive film (First Contact Red, Photonic Cleaning Technologies) was drop‐cast on top of the substrate, allowed to dry at room temperature, and then peeled away by hand, leaving M1 and M2 in a side‐by‐side arrangement and separated by an ODT monolayer. In the final step, the monolayer was removed by O_2_ plasma treatment for 3 min (100 W, O_2_ flow rate: 5 mL min^−1^).

Note, the Al adhesion layer is not essential, and it is possible to fabricate Au‐based nanogap structures without an Al adhesion layer. However, in this case, the strength of adhesion to the underlying substrate is substantially weakened, and the devices must be handled with care to avoid delamination of the gold. Alternatively, the need for a metallic adhesion layer may be avoided by coating the substrate with a thin layer of a polymer such as SU8 prior to deposition of the first metal to ensure adequate metal/substrate adhesion.^[^ ^66^ ^]^

### Fabrication of Multilayer Metal Nanogaps

Fabrication was carried out using an equivalent procedure to the one used for the single‐layer metal nanogaps, except the ODT monolayer was replaced by a molecular ruler, i.e. a metal‐ligated multilayer. The molecular rulers were prepared according to a literature protocol^[^ ^49^ ^]^ by first immersing the substrate in a 2 × 10^−3^ m ethanolic solution of 16‐mercaptohexadecanoic acid (MHDA) for 12 h to form a densely packed monolayer on top of M1 (Au). Further layers of MHDA were then added in a step‐wise manner by alternately immersing the substrate in a 2 × 10^−3^ m ethanolic solution of copper perchlorate for 15 min and a 2 × 10^−3^ m ethanolic solution of MHDA for 30 min, washing thoroughly in clean ethanol between each process step. In the final step of the multilayer preparation (after Cu(ClO_4_)_2_ treatment), the substrate was immersed in a 2 × 10^−3^ m solution of ODT in ethanol for 24 h, yielding an upper surface of non‐reactive alkyl groups in the molecular ruler. From this point onward the fabrication procedure was identical to the single‐layer procedure.

### Fabrication of RSN Arrays

RSN arrays were fabricated according to the single‐ and multilayer nanogap procedures described above, except a template of PS nanospheres was used as a shadow mask for deposition of M1 and 5 nm Ti (instead of 5 nm Al) was deposited beneath the 45 nm gold to minimize plasmonic losses. To prepare the nanosphere template, a glass substrate was sequentially cleaned with acetone, ethanol, and deionized water, dried in a stream of nitrogen, and then subjected to O_2_ plasma for 3 min (100 W, O_2_ flow rate: 5 mL min^−1^). A circular well of polydimethylsiloxane (PDMS) of diameter 1 cm and height 2 mm was placed in the center of the glass substrate. A 10 wt% suspension of 500 nm PS nanospheres in water [Product no. 59769, Sigma Aldrich,] was volumetrically diluted by 50% in ethanol, and loaded into a micropipette. A 0.5 µL droplet of the diluted solution was deposited inside the PDMS well, and allowed to dry under ambient conditions, yielding a close‐packed monolayer of nanospheres. The nanospheres were then etched with O_2_ plasma (100 W, O_2_ flow rate: 5 mL min^−1^) for 10 min, causing them to shrink in volume, while remaining at their original location. A 5 nm adhesion layer of titanium, followed by a 45 nm layer of gold was deposited onto the templated substrate by e‐beam evaporation (10^–7^ mbar, 2 Ås^–1^). The PS nanospheres were removed using one‐sided 3M Scotch tape, leaving behind a hexagonal array of nanoholes in the gold film. The size‐controlled a‐lith method was then carried out using ODT (single layer) or an ODT‐terminated MHDA molecular ruler (multilayer) as described above.

### Imaging

SEM images of the nanogap electrodes were recorded on an electron microscope (FEI APREO) using an electron‐beam voltage of 10 kV and a current of 13 pA. For imaging by TEM, a single‐layer Au/Al nanogap was prepared using the technique described above. The nanogap was then processed into a Au/air/Al laminar using a FIB instrument (FEI Helios) equipped with a nanomanipulator (Omniprobe, AutoProbe300) using the “lift‐out” method. Ion‐beam induced carbon and platinum deposition were performed on the sample surface to protect it against ion‐beam bombardment during ion‐beam milling. A Ga ion‐beam (30 kV, 9 nA) was first used to cut the sample from the bulk, and then it was attached to a Cu grid using the lift‐out method. The sample was subsequently thinned down to a thickness of ≈90 nm (30 kV, 93 pA) for imaging by S(T)EM (S‐5500 model, Hitachi). Prior to FIB patterning, high‐resolution SEM images of the nanogap were recorded on the same S(T)EM using an electron‐beam voltage of 3 kV and a current of 9 pA.

### Electrical Measurements

Molecular diodes based on single and double‐layer Au/Al nanogap electrodes were fabricated by following the single‐ and multilayer procedures mentioned above. The organic spacers were removed by oxygen plasma cleaning for a period of 5 min (100 W, O_2_ flow rate: 5 mL min^−1^). Next, the substrates were immersed in a 5 × 10^−3^ m solution of FcC11 in ethanol for 24 h, rinsed with ethanol, and then dried under nitrogen gas. Electrical contact was made to the devices using a probe station (Micromanipulators, Imina Technologies), before testing in air with a Keithley SCS 4202 parameter analyzer. An equivalent procedure was followed for Au/Au molecular diodes. Forward bias corresponded to the second deposited metal (M2 = Au or Al) being positively biased with respect to the first deposited metal (M1 = Au). Typical fabrication yields for single‐layer devices were 60% or more, with non‐functioning devices exhibiting short‐circuited device characteristics (see Figure S10, Supporting Information).

### Optical Measurements

To prepare the samples for SERS measurements, 5 µL ethanolic solutions of R6G of varying concentration were drop‐cast onto as‐fabricated RSN arrays and dried in air for ≈12 h. Raman spectra were obtained on a confocal Raman spectrometer using 0.5 mW, 633 nm laser excitation. The laser beam was focused onto the sample through a ×50 objective lens (NA = 0.75), and all spectra were acquired using a 10 s acquisition time.

### Simulations

3D electromagnetic simulations were performed with the software package CST Studio in the frequency domain. To simulate the array, periodic boundary conditions were used in the *x*, *y* directions, with a rectangular unit cell of dimensions *P_x_* =  500 nm and *P_y_* = 2 · *P_x_* sin(π/3). The unit cell has one RSN at the center and a quarter of an RSN at each vertex. The thickness of the gold was set to 50 nm for M1 and 30 nm for M2. A linearly polarized plane wave at normal incidence in combination with Floquet Mode Ports was used to simulate the excitation of the λ  =  633 nm laser used for the SERS experiments. The internal diameter of the RSN was 380 nm and the gap width was Δ*r*  =  3 nm, corresponding to *N* = 1. The RSN hexagonal array was simulated on a glass substrate to match the fabricated structure.

## Conflict of Interest

The authors declare no conflict of interest.

## Supporting information

Supporting Information

## Data Availability

Research data are not shared.
