# Supplementary material for: Massively Parallel Arrays of Size‐Controlled Metallic Nanogaps with Gap‐Widths Down to the Sub‐3‐nm Level
Source: Adv Mater. 2021 May 3;33(20):2100491. doi: 10.1002/adma.202100491 (PMC11468177; doi:10.1002/adma.202100491)
Supplement: Supplementary file 1 — Supporting Information [file ADMA-33-2100491-s001.pdf]

© 2021 Wiley-VCH GmbH

ADVANCED MATERIALS

Supporting Information

for *Adv. Mater.,* DOI: 10.1002/adma.202100491

Massively Parallel Arrays of Size-Controlled Metallic Nanogaps with Gap-Widths Down to the Sub-3-nm Level

Sihai Luo, Andrea Mancini, Rodrigo Berté, Bård H. Hoff, Stefan A. Maier, and John C. de Mello**

Supporting Information

Massively parallel arrays of size-controlled metallic nanogaps with gap-widths down to the sub 3-nm level

Sihai Luo[1], Andrea Mancini[2], Rodrigo Berté[2], Bård H. Hoff[1], Stefan A. Maier[2,3] and John C. deMello[1†]*

[1]*Dept. Chemistry, Norwegian University of Science and Technology (NTNU), NO-7491 Trondheim, Norway*
[2]*Nano-Institute Munich, Faculty of Physics, Ludwig-Maximilians-Universität München, 80539 München, Germany*
[3]*Blackett Laboratory, Department of Physics, Imperial College London, London SW7 2AZ, UK*

**sihai.luo@ntnu.no, †john.demello@ntnu.no*

This file includes:

Supplementary Text

Figures S1-S10

Table S1-S3

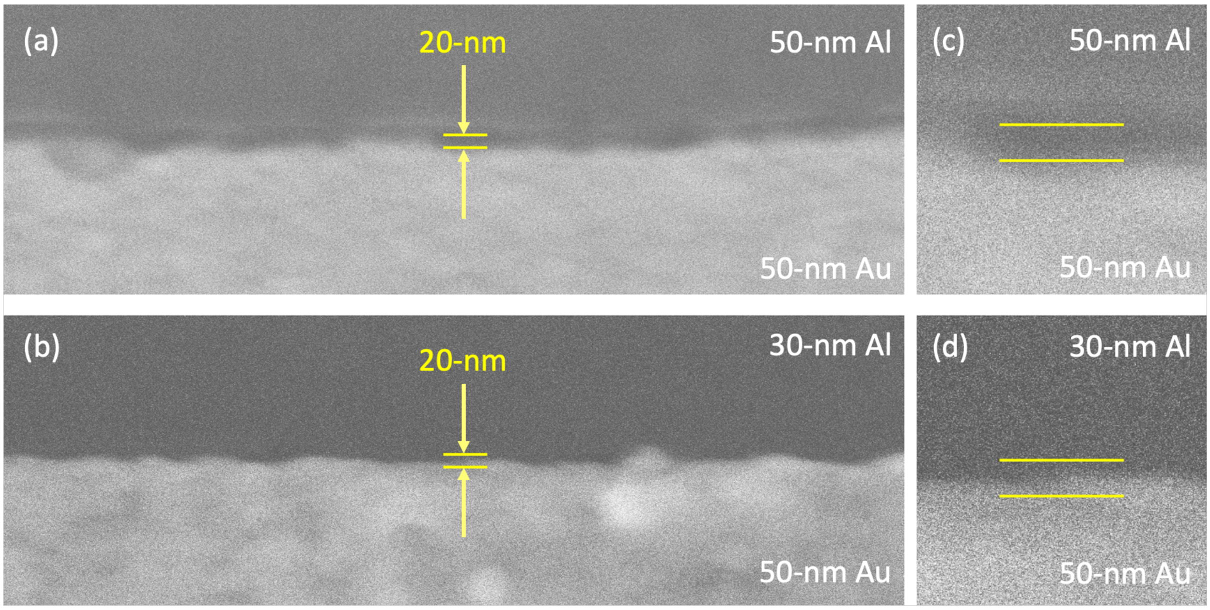

Figure S1. Effect of matched and unmatched metal heights on the gap-width. (a, b) SEM images of Au-Al nanogap electrodes obtained with matched (a) and unmatched (b) metal heights, using ODT as the SAM, 50-nm Au for M1 and either 50 nm Al (a) or 30-nm Al (b) for M2. The horizontal yellow lines are separated by 20 nm. (c, d) Magnified images of the highlighted sections in (a, b). Note, the images shown in this figure are magnified versions of the images in Figure 2a,b (without contrast enhancement). The horizontal yellow lines are separated by 20 nm.

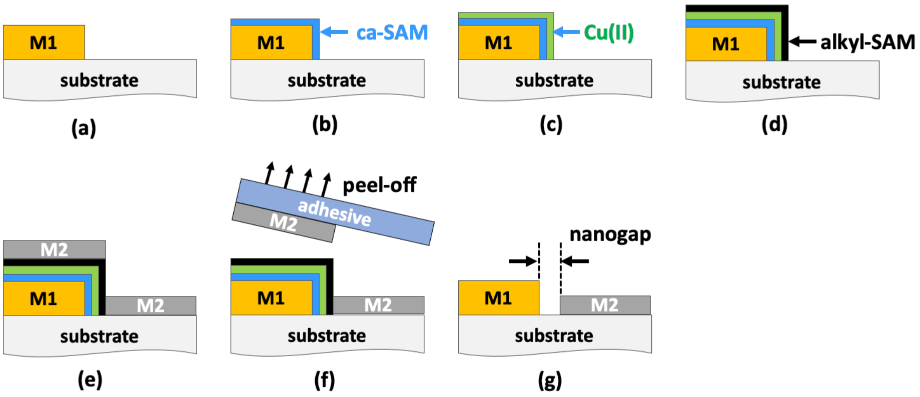

Figure S2. Schematic showing the key processing steps for size-tuneable adhesion lithography (a-g). First, metal M1 is deposited on a substrate and patterned as required (a). Second, M1 is selectively coated with a carboxylic acid-functionalised metallophilic self-assembled monolayer (ca-SAM) (b). Third, the substrate is immersed in a solution of copper perchlorate, causing an atomically thin linker layer of copper ions to attach to the (outwardly facing) carboxylic acid groups on the ca-SAM molecules (c). The second and third-steps may be repeated as required to add further layers to the multilayer. Fourth, for the last layer in the assembly, the multilayer is capped with an alkyl-SAM (d). Fifth, metal M2 is deposited uniformly over the full area of the substrate at a substantially lower thickness than M1 (e). Sixth, an adhesive film is applied to the surface of M2 and peeled away from the substrate, selectively removing M2 from those regions located directly above the multilayer (f). Lastly, the multilayer is removed by UV/ozone or oxygen-plasma treatment, leaving M1 and M2 sitting in a complementary arrangement side-by-side on the substrate (g), separated in the limiting case by the length of the multilayer.

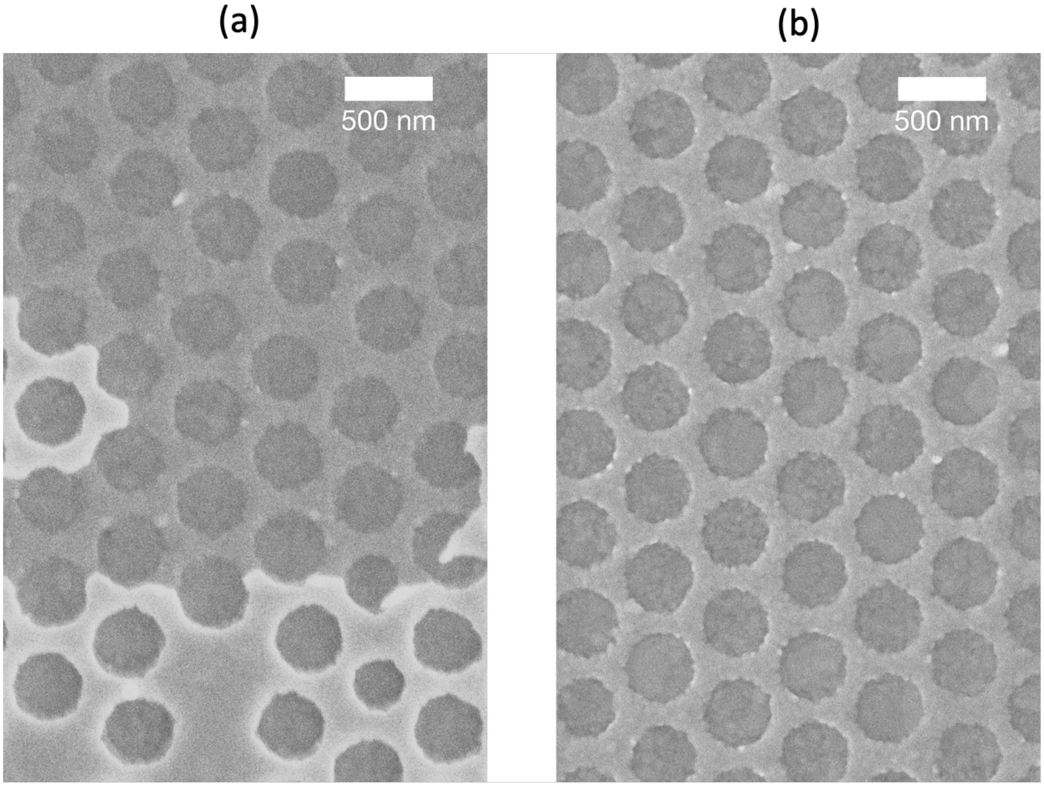

Figure S3. Effect of top-layer SAM on patterning quality. (a) Au/Au nanohole array obtained using MHDA as the top-layer, showing incomplete removal of the unwanted parts of the second metal M2. The bright regions correspond to unwanted gold that should have been stripped away but has instead been retained due to excessive adhesion between MHDA and Au. (b) Equivalent Au/Au nanohole array obtained using ODT as the top-layer, showing successful removal of the unwanted parts of the second metal M2. Arrays have a pitch of ~500 nm and a ring-diameter of ~380 nm, defined by the nanosphere diameters before and after etching.

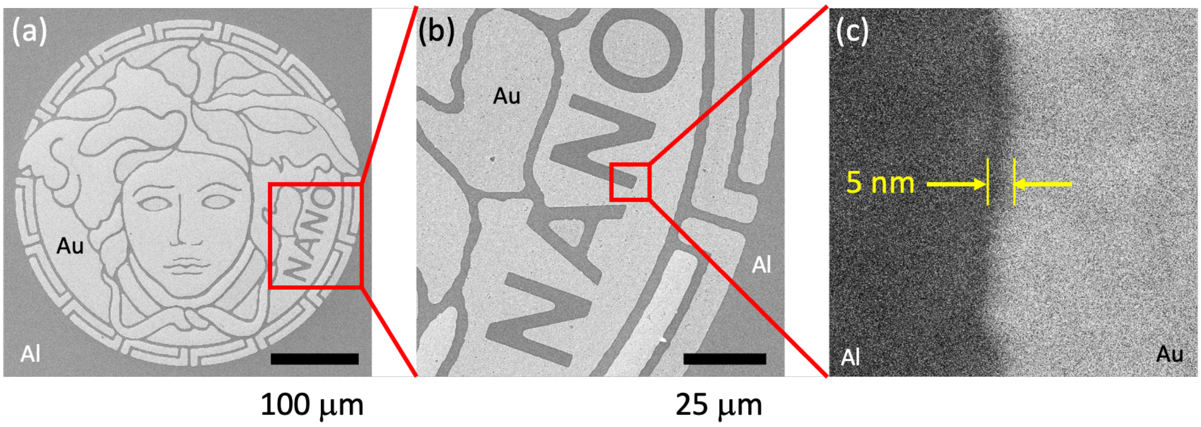

Figure S4. Multi-scale patterning by adhesion lithography. (a) Low magnification scanning electron micrograph of "Medusa", fabricated in gold and aluminium by adhesion lithography using a single layer of ODT. (b) Magnified section of the micrograph from (a). (c) Representative SEM image for a section of the Al/Au interface inside the red box in (b). A sub 5-nm nanogap exists at the interface between the two metals despite the full pattern extending over hundreds of microns.

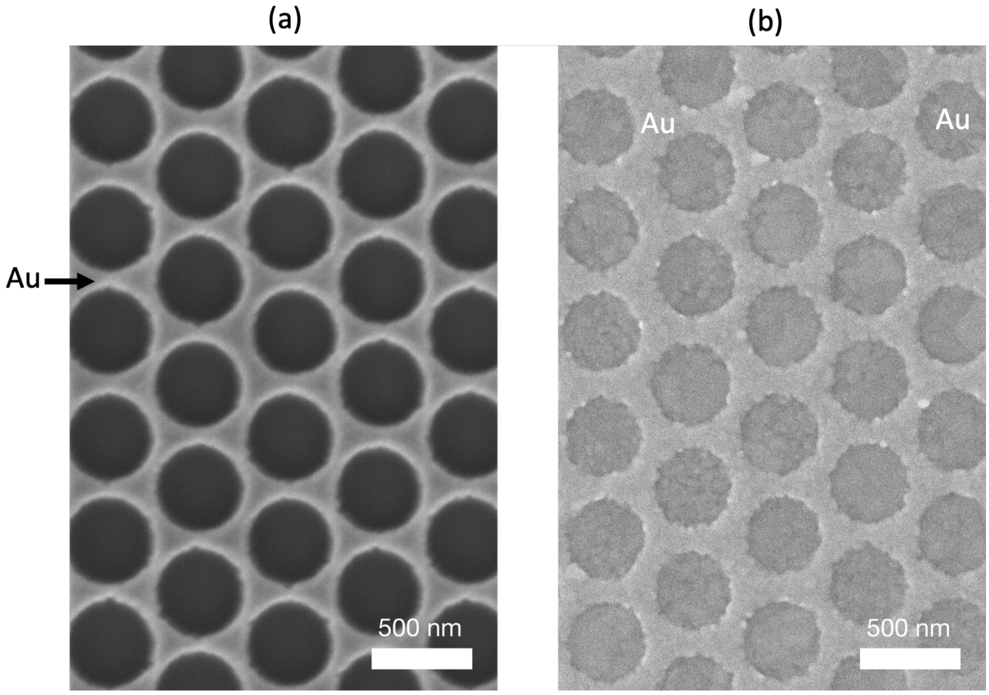

Figure S5. Nanohole and nanoring arrays, obtained using a combination of nanosphere lithography and adhesion lithography. (a) Nanohole array obtained by terminating fabrication after removal of the nanosphere template, i.e. at step (c) in Figure 4 of the main article. Dark regions indicate the exposed substrate (glass). (b) Nanoring array obtained by carrying out the fabrication process to completion, i.e. to step (d) in Figure 4. Arrays have a pitch of ~500 nm and a ring-diameter of ~380 nm, defined by the nanosphere diameters before and after etching. A single layer of ODT was used as the adhesion modifier.

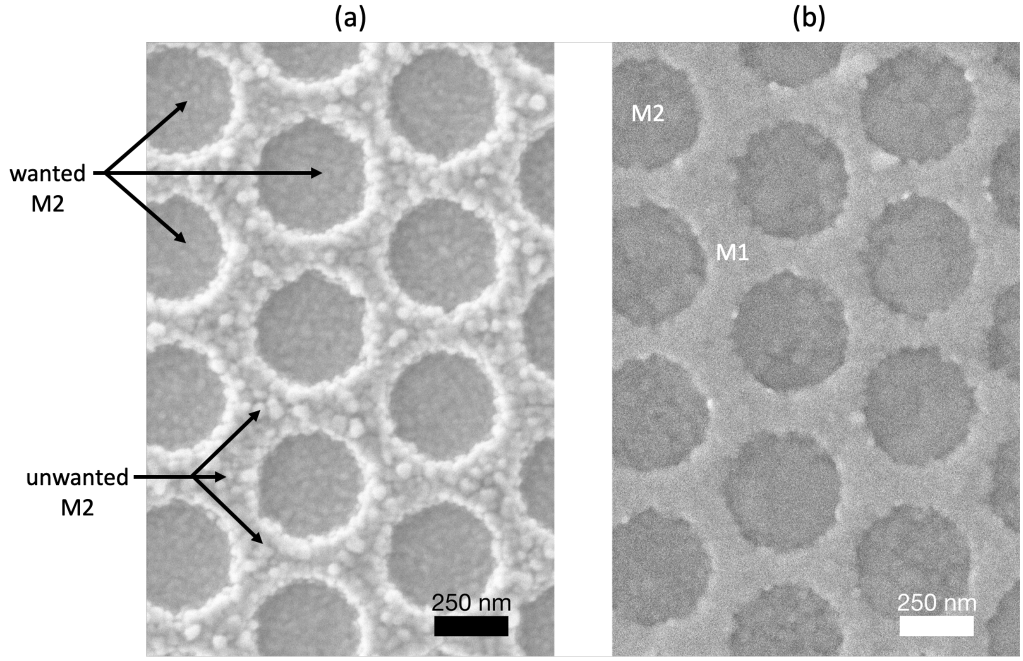

Figure S6. Au/Au nanoring arrays before and after the peeling step. Prior to peeling (a), a clear height difference is evident between the wanted (dark, recessed) and unwanted (bright, protruding) parts of M2, causing pre-fracturing along the edge profile of M1. After peeling (b), M2 is recessed with respect to M1 due to its lower thickness. Arrays have a pitch of ~500 nm and a ring-diameter of ~380 nm, defined by the nanosphere diameters before and after etching. ODT was used as an adhesion modifier.

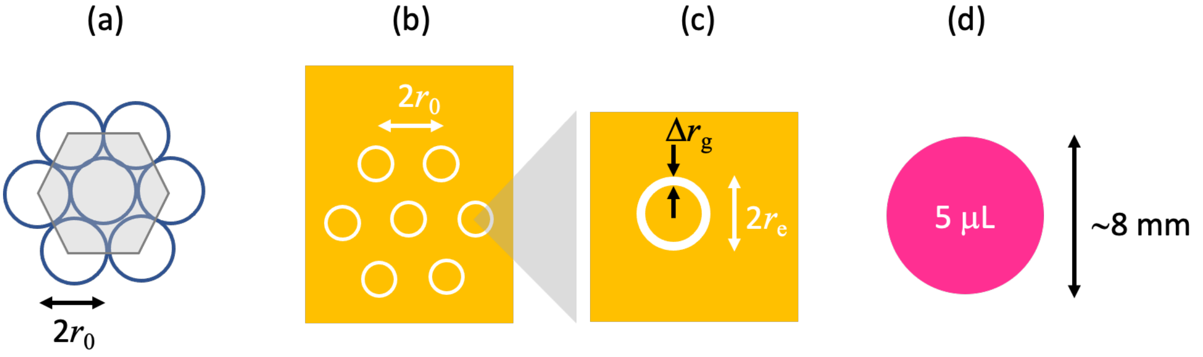

Figure S7. (a) Schematic showing polystyrene (PS) nanospheres of radius r_0 in a hexagonal close-packed arrangement. The hexagonal unit cell containing a total of three spheres is shown in grey. (b) Schematic of ring-shaped nanogap array, with a pitch $2r_0$ that is determined by the original (unetched) radius r_0 of the PS nanospheres. (c) 'Close-up' schematic of individual nanoring of diameter $2r_e$ and gap-width Δr_g, where r_e is the radius of the etched PS nanospheres and Δr_g is determined by the number of layers in the molecular ruler. (d) A 5-µL drop-cast dye solution fills a roughly circular spot of diameter 8 mm. From simple geometry, the area A of the hexagonal unit cell in (a) is given by $A = 6\sqrt{3}r_0^2$. Hence, the area – inclusive of voids – A_0 per sphere (or equivalently the area per ring in the final nanogap array) is one third this value, i.e. $A_0 = 2\sqrt{3}r_0^2$. The approximate area A_g of each ring-shaped gap is given by $A_g = 2\pi r_e \Delta r_g$. Hence, the fraction F of the array area that is covered by nanogaps is given by $F = A_g/A_0 = (\pi/\sqrt{3})\Delta r_g(r_e/r_0^2)$. Putting $r_0 = 250$ nm, $r_e = 190$ nm and $\Delta r_g = 3$ nm, we obtain $A_0 = 216506$ nm^2, $A_g = 3581$ nm^2 and $F = 1.65$ %. A 5-µL drop of Rhodamine 6G dye solution deposited on the nanogap array forms a roughly circular spot of diameter 8 mm, corresponding to an approximate area $A_{dye} = 50$ mm^2 that covers around 230 million nanorings ($N_{ring} = A_{dye}/A_0$). At the lowest detectable dye concentration of $c = 10^{-14}$ M, a drop of volume $V = 5$ µL contains around 30000 dye molecules ($N_{avg}cV$). For a random arrangement of dye molecules on top of the nanoring array, approximately 500 molecules are centred inside the nanogap ($FN_{avg}cV$), implying the observed Raman scattering signal is due to of order 1000 dye molecules.

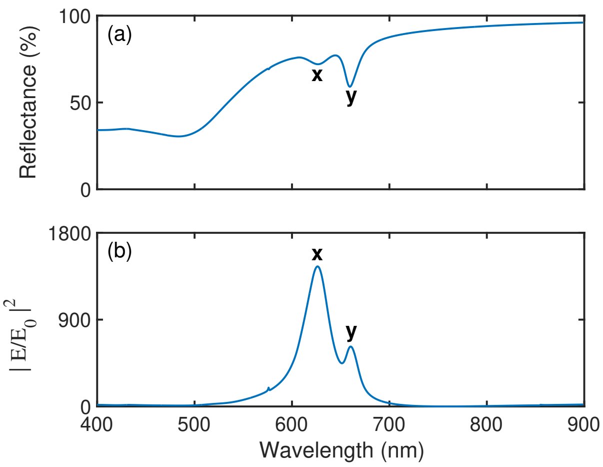

Figure S8. Simulation of the optical response of a Au/Au nanogap array with 3-nm gap-width. Simulated reflectance spectra (a) and field enhancement ($|E/E_0|^2$) spectra (b), determined at the centre of the gap and level with the upper surface of M2. Calculations were carried out for an array pitch of 500 nm, a ring diameter of 380 nm, a gap-width of 3 nm and heights of 50 and 30 nm for M1 and M2, respectively. The dip in the reflectance spectrum at λ = 626 nm is due to the dipole mode arising across the edges of the gap, while the dip at λ=660 nm is an array mode. The frequency of the gap resonance (x) is mainly sensitive to the width of the gap, while the frequency of the array resonance (y) is mainly sensitive to the pitch of the array. The drop in reflectivity for wavelengths below 600 nm is due to the interband transition of gold. Even though the array resonance gives rise to a larger dip in the reflectance spectrum, it can be seen from (b) that the gap resonance at λ = 626 nm is more effective at concentrating the electromagnetic energy inside the gap region, leading to higher SERS enhancement. The broad width of the gap-resonance results in strong SERS enhancement even when the excitation wavelength differs from the peak resonance by several tens of nanometres.

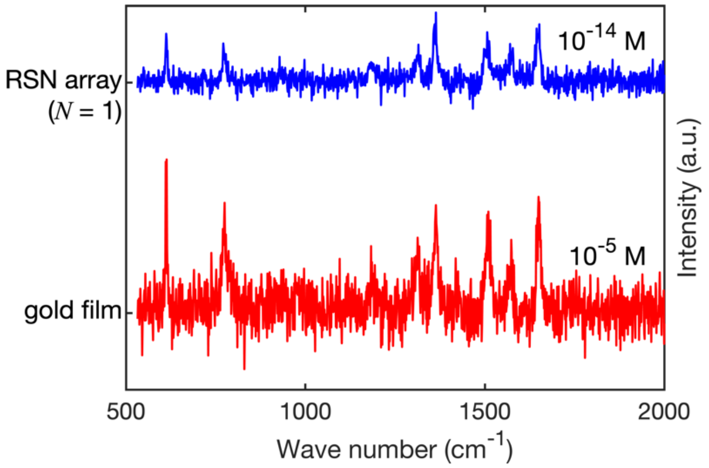

Figure S9. Determination of the Raman scattering enhancement factor relative to a thin gold film. The blue trace shows a Raman scattering spectrum for Rhodamine 6G drop-cast from a 10^{-14} M solution onto an Au-Au RSN array with a pitch of ~500 nm and a ring-diameter of ~380 nm, fabricated using a molecular ruler of length $N = 1$ (see Figure 4f). The red trace shows a Raman scattering spectrum for Rhodamine 6G drop-cast from a 10^{-5} M solution onto a 50-nm gold film. 10^{-14} M and 10^{-5} M represent the Raman scattering detection limits for the RSN array and the gold film, respectively. Spectra were obtained using identical acquisition parameters, see Experimental section. Following Ref. S1, the analytical enhancement factor γ at 613 cm^{-1} was determined using:

$$\gamma = \frac{I_{\mathrm{RSN}}(613\ \mathrm{cm}^{-1})/10^{-14}}{I_{\mathrm{Au}}(613\ \mathrm{cm}^{-1})/10^{-5}} = \frac{0.3185/10^{-14}}{1.0/10^{-5}} \approx 3 \times 10^{8}.$$

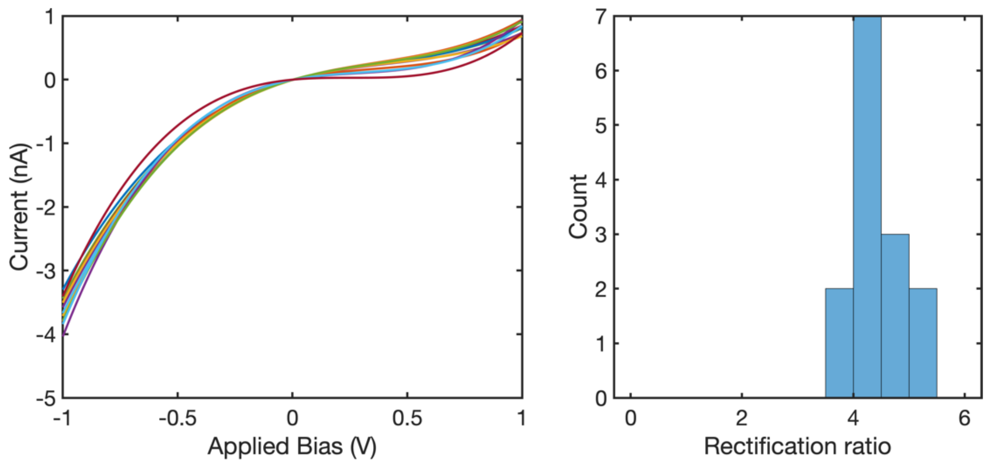

Figure S10. Electrical characteristics for fourteen (out of twenty two) nominally identical Au/FcC11/Al molecular diodes fabricated using molecular rulers of length *N* = 1, see Fig. 3a for device structure. (a) *I-V* characteristics for the devices The remaining 8 of the 22 devices were short-circuited. (b) Histogram showing the distribution of rectification ratios for the functioning devices, measured at ±1 V.

Table S1 Characteristics of common nanogap fabrication techniques.[S2, S3] The term "large-area" indicates that the fabrication technique is compatible with wafer-scale processing.

	Method	Resolution (nm)	Large-area? Yes / No	Throughput*	Parallel? Yes / No	Cost*
**Optical**	Photolithography (EUVL)	~10 [S4]	Y	H	Y	H
	Laser-direct writing	~5 [S5]	Y	H	Y	M
**Beam**	E-beam lithography	~5 [S6]	Y	M	N	M
	FIB milling	~3 [S7]	N	M	N	H
	TEM milling	< 1 [S8]	N	L	N	H
**Breaking or Cracking**	Mechanically controlled breaking	~1 [S9]	N	L	N	H
	Crack junction	< 3 [S10]	Y	M	Y	M
	Electromigration	~1 [S11]	N	L	N	M
**Peeling**	Atomic layer lithography	< 1 [S12]	Y	H	Y	M
	'Sketch and peel'	~5 [S13]	Y	M	Y	H
	a-lith	< 3 [This work]	Y	H	Y	L

*L = Low, M = Medium, H = High

Table S2 Measured film thickness and nanogap widths for monolayers and molecular rulers of varying length. Film thicknesses were measured using an ellipsometer (J.A. Wollam M-2000), while gap-widths were extracted from the SEM images in Figure 2.

**Molecular Ruler**	**No. layers**	**Thickness (nm)**	
**$[HS(CH_2)_{15}COOH]_n[HS(CH_2)_{17}CH_3]_m$**	**(n + m)**	**Film**	**Nanogap**
n = 0, m = 1 (MHDA monolayer)	1	2.2 ± 0.2	2.8 ± 1.1
n = 1, m = 0 (ODT monolayer)	1	2.1 ± 0.2	NA
n = 1, m = 1	2	4.5 ± 0.3	4.6 ± 1.7
n = 4, m = 1	5	11.8 ± 0.3	13.2 ± 2.7
n = 9, m = 1	10	22.6 ± 0.3	21.3 ± 3.3
n = 15, m = 1	16	33.2 ± 0.2	30.7 ± 4.2

Table S3 Fabrication methods for SERS substrates and reported SERS activities using rhodamine 6g

**Ref.**	**Fabrication method**	**Structure**	**Characteristics**
[S14]	Nanosphere lithography	"Lotus seed pods"	EF: 1.4 X 10^7<br>Detection limit: 10^{-12} M
[S15]	Nanosphere lithography, chemical etching	Si nanorod arrays functionalised with Au nanoparticles	EF: 3.3 X 10^7<br>Detection limit: 10^{-10} M
[S16]	Solvent-assisted nanotransfer printing	"3D cross-point plasmonic nanoarchitectures"	EF: 4.1 X 10^7<br>Detection limit: NA
[S17]	Controlled evaporation deposition process	Vertically aligned Au nanorods	EF: NA<br>Detection limit: 10^{-15} M
[S18]	Binary-template-assisted electrodeposition	Ag nanorod bundles	EF: 1.4 X 10^8<br>Detection limit: 10^{-9} M
[S19]	Nanosphere lithography, reactive ion etching	Hierarchical Ag nanocone arrays	EF: NA<br>Detection limit: 10^{-17} M
[S20]	Block copolymer lithography	Au-Ag core-shell nanoparticle arrays	EF: NA<br>Detection limit: 10^{-8} M
[S21]	Solution-phase synthesis	Au nanostar arrays	EF: 4.5 X 10^8<br>Detection limit: NA
This work	Size-tuneable adhesion lithography	Au nanoring arrays with sub-3-nm gap	EF: 3 X 10^8<br>Detection limit: 10^{-14} M

References

[S1] E. C. Le Ru, E. Blackie, M. Meyer, P. G. Etchegoint, *J. Phys. Chem. C.* **2007**, *111*, 13794.
[S2] V. Dubois, S. J. Bleiker, G. Stemme, F. Niklaus, *Adv. Mater.* **2018**, *30*, 1801124.
[S3] Y. Yang, C. Gu, J. Li, *Small* **2019**, *15*, 1804177.
[S4] T. Siegfried, Y. Ekinci, H. H. Solak, O. J. F. Martin, H. Sigg, *Appl. Phys. Lett.* **2011**, *99*, 2009.
[S5] L. Qin, Y. Huang, F. Xia, L. Wang, J. Ning, H. Chen, X. Wang, W. Zhang, Y. Peng, Q. Liu, Z. Zhang, *Nano Lett.* **2020**, *20*, 4916.
[S6] M. Chirumamilla, A. Toma, A. Gopalakrishnan, G. Das, R. P. Zaccaria, R. Krahne, E. Rondanina, M. Leoncini, C. Liberale, F. De Angelis, E. Di Fabrizio, *Adv. Mater.* **2014**, *26*, 2353.
[S7] M. K. Kim, H. Sim, S. J. Yoon, S. H. Gong, C. W. Ahn, Y. H. Cho, Y. H. Lee, *Nano Lett.* **2015**, *15*, 4102.
[S8] M. D. Fischbein, M. Drndić, *Nano Lett.* **2007**, *7*, 1329.
[S9] M. L. Perrin, C. J. O. Verzijl, C. A. Martin, A. J. Shaikh, R. Eelkema, J. H. Van Esch, J. M. Van Ruitenbeek, J. M. Thijssen, H. S. J. Van Der Zant, D. Dulić, *Nat. Nanotechnol.* **2013**, *8*, 282.
[S10] V. Dubois, S. N. Raja, P. Gehring, S. Caneva, H. S. J. van der Zant, F. Niklaus, G. Stemme, *Nat. Commun.* **2018**, *9*, 3433.
[S11] Y. Naitoh, T. Ohata, R. Matsushita, E. Okawa, M. Horikawa, M. Oyama, M. Mukaida, D. F. Wang, M. Kiguchi, K. Tsukagoshi, T. Ishida, *ACS Appl. Mater. Interfaces* **2013**, *5*, 12869.
[S12] X. Chen, H. R. Park, M. Pelton, X. Piao, N. C. Lindquist, H. Im, Y. J. Kim, J. S. Ahn, K. J. Ahn, N. Park, D. S. Kim, S. H. Oh, *Nat. Commun.* **2013**, *4*, 2361.
[S13] Y. Chen, Q. Xiang, Z. Li, Y. Wang, Y. Meng, H. Duan, *Nano Lett.* **2016**, *16*, 3253.
[S14] B. Jin, J. He, J. Li, Y. Zhang, *Adv. Opt. Mater.* **2018**, *6*, 8.
[S15] D. Lin, Z. Wu, S. Li, W. Zhao, C. Ma, J. Wang, Z. Jiang, Z. Zhong, Y. Zheng, X. Yang, *ACS Nano*. **2017**, *11*, 1478.
[S16] J. W. Jeong, M. M. P. Arnob, K.-M. Baek, S. Y. Lee, W.-C. Shih, Y. S. Jung, *Adv. Mater.* **2016**, *28*, 8695.
[S17] W. Wei, Y. Wang, J. Ji, S. Zuo, W. Li, F. Bai, H. Fan, *Nano Lett.* **2018**, *18*, 4467.
[S18] C. Zhu, G. Meng, P. Zheng, Q. Huang, Z. Li, X. Hu, X. Wang, Z. Huang, F. Li, N. Wu, *Adv. Mater.* **2016**, *28*, 4870.
[S19] Y. Guan, Z. Wang, P. Gu, Y. Wang, W. Zhang, G. Zhang, *Nanoscale*. **2019**, *11*, 9422.
[S20] S. K. Cha, J. H. Mun, T. Chang, S. Y. Kim, J. Y. Kim, H. M. Jin, J. Y. Lee, J. Shin, K. H. Kim, S. O. Kim, *ACS Nano*. **2015**, *9*, 5536.
[S21] W. Niu, Y. A. A. Chua, W. Zhang, H. Huang, X. Lu, *J. Am. Chem. Soc.* **2015**, *137*, 10460.
